# Gallstone ileus presenting in an elderly patient: A case report

**DOI:** 10.1016/j.ijscr.2024.110440

**Published:** 2024-10-12

**Authors:** María Elisa González-Robles, Laís Lorena Menéndez-Goti, Antonio de Jesús González-Luna, Cristina Vanessa Cuevas-Calla, Quitzia Libertad Torres-Salazar

**Affiliations:** a Guadalajara Civil Hospital, Mexico; b Autonomous University of Guadalajara, Mexico; c Regional Hospital “Dr. Valentín Gómez Farias”, Institute of Security and Social Services for State Workers, Mexico; d Juárez University of the State of Durango, Mexico

**Keywords:** Biliary ileus, Gastric surgery, Acute abdomen, Obstruction

## Abstract

**Introduction and importance:**

Biliary ileus is a rare yet significant cause of mechanical intestinal obstruction, which occurs when a gallstone enters the gastrointestinal tract through a bilioenteric fistula, leading to intestinal blockage. This condition primarily affects elderly patients and is associated with high morbidity and mortality if not diagnosed and treated promptly.

**Case presentation:**

We present the case of a 94-year-old female with a history of hypertension and chronic venous insufficiency. The patient was admitted with severe abdominal pain, nausea, and vomiting, with clinical findings suggestive of intestinal obstruction. Computed tomography revealed Rigler's triad, confirming the diagnosis of biliary ileus. An exploratory laparotomy was performed, identifying three gallstones in the small intestine. The patient underwent enterotomy for stone extraction and had a favorable immediate postoperative outcome.

**Clinical discussion:**

Biliary ileus presents a diagnostic challenge due to its nonspecific symptoms. While Rigler's triad (pneumobilia, intestinal obstruction, and ectopic gallstone) is diagnostic, it is not always apparent in imaging. Surgical intervention remains the standard of care for resolving the obstruction, though appropriate preoperative management and timely surgery are crucial for improving outcomes.

**Conclusion:**

This case emphasizes the importance of considering biliary ileus in the differential diagnosis of intestinal obstruction, particularly in elderly patients. Early surgical intervention is essential to prevent severe complications.

Evidence based medicine ranking: Level IV.

## Highlights

• Biliary ileus is a rare yet significant cause of mechanical intestinal obstruction.
• Diagnosis is confirmed by Rigler's triad.
• Timely surgical intervention is crucial to prevent severe complications.

## 1 Introduction

Biliary ileus is a rare cause of intestinal obstruction, accounting for approximately 0.5–4 % of all cases of obstruction but up to 25 % in patients over 65 years of age [1]. The pathogenesis of biliary ileus involves the formation of a fistula between the gallbladder and the gastrointestinal tract, allowing gallstones to enter the intestine, where they can cause obstruction. The signs and symptoms of biliary ileus are nonspecific; however, 40–50 % of patients may have a history of biliary colic, jaundice, or acute cholecystitis. The most common site of gallstone impaction is the distal ileum (60 %), due to its narrower lumen and decreased peristaltic activity [2]. Although Rigler's triad (pneumobilia, intestinal obstruction, and ectopic gallstone) is diagnostic, it is not always visible on imaging, making diagnosis challenging. Surgical treatment, typically involving stone extraction and fistula repair, if possible, remains the standard approach [3]. Given that biliary ileus is a rare but clinically relevant entity, particularly in elderly patients, timely diagnosis can be challenging due to its nonspecific symptoms and inconsistent visibility of Rigler's triad in radiologic images. Presenting a case of biliary ileus not only enhances our understanding of this pathology but also underscores the importance of clinical suspicion and appropriate surgical management in vulnerable populations. This study adds several important and novel contributions to the current understanding and management of biliary ileus. First, it highlights the diagnostic complexities encountered in elderly patients due to nonspecific symptoms and inconsistent presentation of classical radiographic findings, emphasizing the crucial role of computed tomography (CT) imaging in securing an accurate diagnosis. Second, the study provides valuable insight into the benefits of a conservative surgical approach—specifically, performing an enterotomy without a cholecystectomy in high-risk patients. By illustrating the safety and efficacy of this approach in individuals with significant comorbidities, this report supports the evolving “less is more” philosophy aimed at minimizing surgical morbidity while ensuring favorable outcomes. Furthermore, the case advocates for individualized treatment strategies based on patient stability and surgical risk, promoting a tailored approach that prioritizes patient safety and outcomes, particularly in high-risk elderly populations. These contributions underscore the necessity for flexible decision-making in complex surgical scenarios, providing a more nuanced understanding of biliary ileus management, especially within a vulnerable demographic. This report follows the SCARE criteria [4].

## 2 Clinical case presentation

We present the case of a 94-year-old female with a history of hypertension and chronic venous insufficiency. The patient was admitted to the emergency department of a hospital in Tijuana, Baja California, with a clinical presentation of severe abdominal pain localized to the right upper quadrant, accompanied by persistent nausea, vomiting, and abdominal distension. These symptoms had gradually developed over two days but significantly worsened within the last 24 h. The patient also reported a history of chronic constipation, although she had not had any bowel movements for five days. The patient had no previous surgeries.

On initial physical examination, the patient was afebrile but exhibited signs of dehydration and mild tachycardia. Abdominal palpation revealed tenderness in the upper abdomen, with moderate guarding and absent bowel sounds. A rectal examination showed no significant findings. Given the clinical characteristics, intestinal obstruction was suspected, and imaging studies were ordered to confirm the diagnosis.

**Fig. 1:**
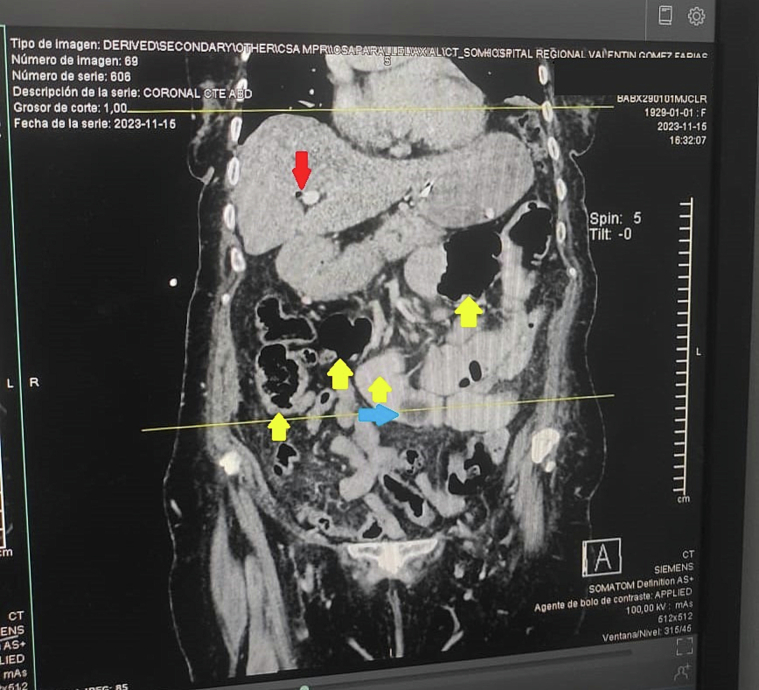
Shows a coronal view of a computed tomography scan of the abdomen, revealing multiple distended intestinal loops, suggestive of an intestinal obstruction. Additionally, pneumobilia is clearly visible, which is a key finding in the diagnosis of biliary ileus. These features are consistent with Rigler's triad, which includes intestinal obstruction, pneumobilia, and the presence of an ectopic gallstone. Red Arrow: Indicates pneumobilia, which is the presence of air within the biliary tract. Air should not be present in this location unless the patient has undergone an endoscopic procedure, such as an ERCP, or has a history of prior surgery. The presence of air suggests a communication between the biliary system and a segment of the intestine, allowing intestinal air to enter the biliary ducts. Blue Arrow: Highlights the density of the gallstone causing intestinal obstruction. Yellow Arrows: Represent the intestinal obstruction located proximally to the gallstone causing the blockage. (For interpretation of the references to colour in this figure legend, the reader is referred to the web version of this article.)

A plain abdominal radiograph showed air-fluid levels in the intestinal loops, consistent with obstruction. Contrast-enhanced computed tomography (CT) revealed Rigler's triad: pneumobilia, dilated intestinal loops, and a 3 cm gallstone in the lumen of the small intestine, confirming the diagnosis of biliary ileus. The bilioenteric fistula was not clearly visualized in the imaging (Figs. 1). The hospital did not have a resonator, so magnetic resonance imaging (MRI) could not be performed, though CT remains the study of choice in such cases.

**Fig. 2:**
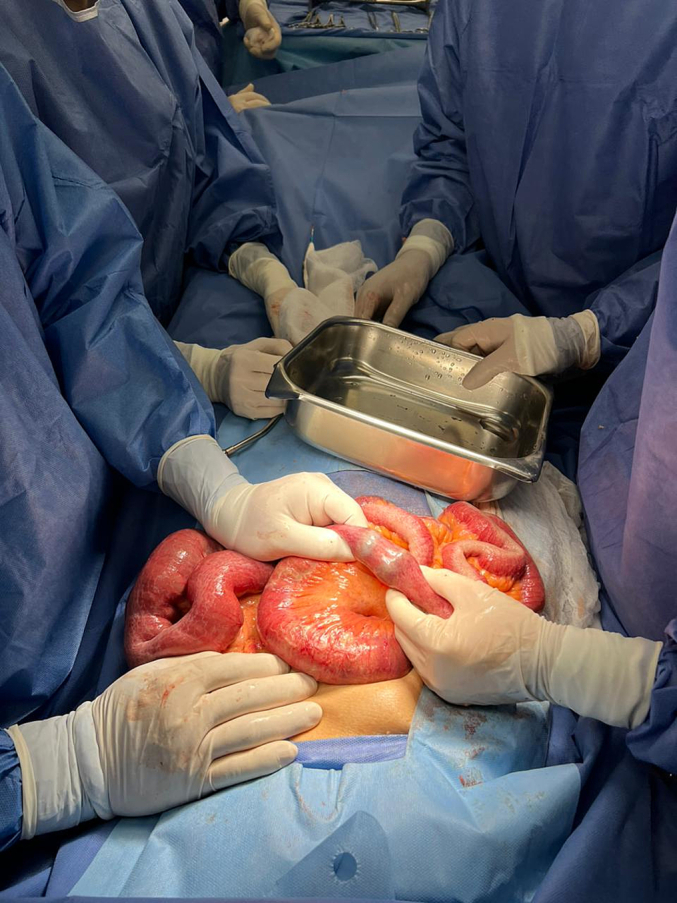
Displays small bowel loops containing gallstones, which have resulted in an intestinal obstruction. These findings are consistent with a diagnosis of biliary ileus.

**Fig. 3:**
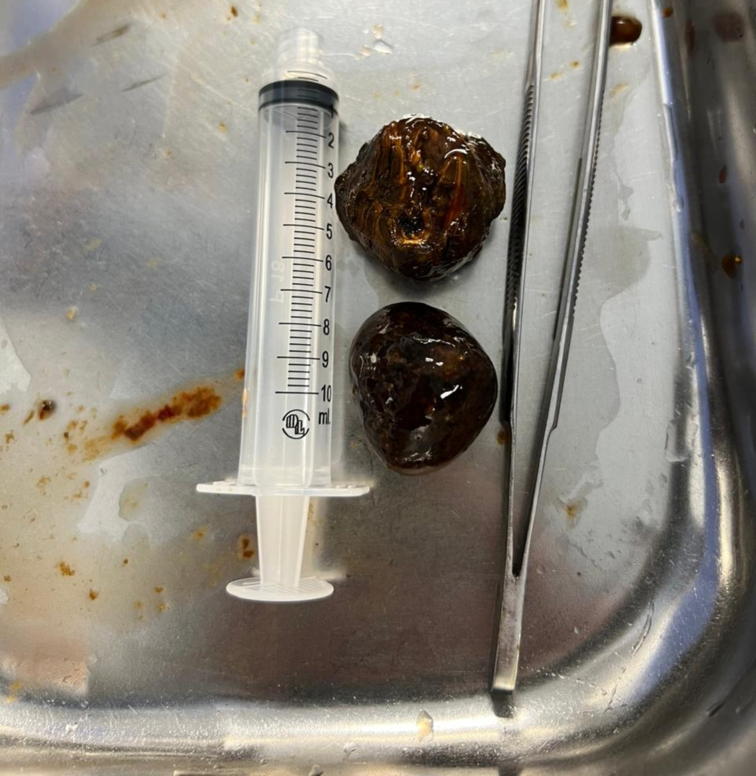
Shows larger gallstones. The pathology report indicates the dimensions are 7.5 × 4 × 2.5 cm.

Due to the severity of the condition and the risk of further complications, emergency surgery was performed. During the exploratory laparotomy, an area of obstruction was identified in the distal ileum (Fig. 2). Upon opening the intestinal lumen, three gallstones were found, the largest measuring 3 cm in diameter, and were successfully removed through enterotomy (Fig. 3). No evident bilioenteric fistula was identified, and a cholecystectomy was not performed due to the patient's stable hemodynamic status and intraoperative assessment.

The surgical procedure began with a midline infra- and supraumbilical incision of approximately 20 cm, providing adequate access to the abdominal cavity. A systematic exploration of the cavity was performed, identifying three gallstones located in the small intestine, 160 cm from the Treitz ligament and 280 cm from the ileocecal valve. Subsequently, a longitudinal enterotomy of 3 cm was performed on the small intestine to extract the gallstones. The enterotomy was closed in two layers using 3–0 silk, first with a continuous suture in the mucosa and then with Lembert sutures, ensuring adequate closure to prevent leaks. During the procedure, the gallbladder was palpated, revealing facet-shaped stones similar to those extracted from the intestine; however, a cholecystectomy was not performed due to the risk of complications, such as duodenal fistula formation. After stone extraction, a thorough lavage of the abdominal cavity was performed, and a Jackson-Pratt drain was placed for postoperative monitoring. Finally, the surgical wound was closed in layers using 1–0 vicryl and 3–0 nylon to ensure tissue integrity.

The immediate postoperative period was uneventful. The patient was monitored in the intermediate care unit for the first 48 h. A clear liquid diet was gradually introduced, and the patient tolerated oral intake by the fourth postoperative day. On the seventh day, she was discharged with scheduled outpatient follow-up. At the first follow-up visit two weeks post-surgery, the patient reported significant improvement in symptoms, with no recurrence of abdominal pain or nausea. Continued regular follow-up and additional imaging studies were recommended to monitor the biliary tract.

## 3 Discussion

Biliary ileus, though a rare cause of intestinal obstruction, continues to present a significant diagnostic challenge, particularly in elderly patients. In our case, a 94-year-old female exhibited clinical signs of intestinal obstruction, which led to the diagnosis of biliary ileus through computed tomography, confirming Rigler's triad (pneumobilia, intestinal obstruction, and ectopic gallstone). However, Rigler's triad is not always evident, making the diagnosis challenging in many instances. Studies have demonstrated that CT with contrast is the preferred diagnostic tool due to its high sensitivity, which can reach over 90 %, while plain abdominal radiographs alone may have a lower diagnostic accuracy [5,6]. For instance, Da Cunha et al. indicated that CT scans successfully confirmed the diagnosis in 81 % of colonic gallstone ileus cases, emphasizing its utility as a primary diagnostic modality [7].

When comparing our case to others reported in the literature, we find similarities in diagnostic challenges and management options. For example, Boudou et al. also emphasized the difficulty of diagnosing biliary ileus due to the nonspecific nature of its symptoms and the associated diagnostic delays [8]. In both our case and others in the literature, computed tomography was a key diagnostic tool, consistent with findings from Vera-Mansilla et al., who reported that over half of the patients were diagnosed preoperatively through imaging, thereby avoiding the need for emergent intraoperative diagnosis [6]. However, unlike our case, where the bilioenteric fistula was not clearly visualized, Boudou et al. identified a cholecystoduodenal fistula, which allowed for more targeted surgical management. This highlights how the visualization of the fistula can influence surgical decisions, potentially improving outcomes.

Another crucial aspect is the surgical approach. In our case, we opted for an enterotomy with gallstone extraction without performing a cholecystectomy due to the risk of complications. Similarly, Helmy et al. reported cases of biliary ileus post-cholecystectomy where a similar surgical approach was taken, prioritizing stone extraction over immediate fistula repair [9]. This less invasive approach is also supported by Xue et al., who note that enterotomy alone may be associated with lower mortality rates and better outcomes compared to more extensive surgical techniques [10]. Vera-Mansilla et al. further supported this, finding that 83 % of patients treated solely with enterolithotomy did not experience any cholecystobiliary complications during follow-up, suggesting that additional procedures such as urgent or delayed cholecystectomy may not be necessary in most cases [6]. The focus on the “less is more” principle is also evident in the work of Reyes-Morales, which underscores enterolithotomy as the recommended procedure due to its lower associated morbidity and mortality [5].

The decision to forego cholecystectomy during the initial surgery aligns with the findings from multiple studies indicating that, for elderly patients or those with significant comorbidities, avoiding extensive procedures helps minimize operative risks and reduce mortality [6]. In a retrospective review, Vera-Mansilla et al. concluded that enterolithotomy without further biliary intervention was a safe and effective treatment for biliary ileus, with a low rate of subsequent biliary complications. These outcomes suggest that in patients with stable hemodynamics and high surgical risk, the primary focus should be on relieving the obstruction with minimal intervention.

Regarding the location of the gallstone, our case is consistent with the majority of reports in the literature, which cite the distal ileum as the most common site of gallstone impaction due to its narrower lumen and reduced peristaltic activity [2]. This finding aligns with those of multiple studies, including the review by Vera-Mansilla et al., who reported a 64 % occurrence of gallstone impaction in the ileum among their cohort [6]. However, other authors, such as Da Cunha et al., or Xue P et al., have reported impactions in less common sites like the colon, where the surgical approach must be modified based on anatomical considerations. In the case of colonic impaction, less invasive approaches like mechanical lithotripsy or endoscopy may be attempted initially, though surgery remains the mainstay of treatment for most cases due to inconsistent outcomes with these newer techniques [7,10].

This case underscores the importance of early diagnosis and timely surgical intervention in patients with biliary ileus, especially in elderly populations [1,8]. The mortality associated with biliary ileus can be reduced significantly by prioritizing rapid diagnosis and adopting a tailored surgical approach. While our surgical approach aligned with the common practice of performing enterotomy with stone extraction, variations in clinical presentation and the decision to repair the bilioenteric fistula highlight the need to individualize treatment based on the patient's clinical condition and intraoperative findings. The recommendation for enterolithotomy alone, supported by the work of Vera-Mansilla and Reyes-Morales, emphasizes the benefit of a less invasive approach whenever possible, given the complexity and heterogeneous nature of biliary ileus [5,6].

## 4 Conclusions

In conclusion, this article presents novel contributions to the understanding and management of biliary ileus, particularly in elderly patients with intestinal obstruction. The case report highlights the diagnostic challenges arising from nonspecific clinical presentations and the inconsistent visualization of Rigler's triad, emphasizing the pivotal role of CT imaging in achieving diagnostic accuracy. Furthermore, the findings underscore the value of a conservative surgical approach—specifically, enterotomy without cholecystectomy—in patients with high surgical risk, thus supporting the evolving “less is more” philosophy in surgical practice. By advocating for individualized treatment strategies based on patient stability and surgical risk, this study contributes to optimizing patient outcomes while minimizing morbidity and mortality.

Moreover, the insights provided by this case reinforce the importance of early diagnosis and tailored therapeutic interventions in the management of biliary ileus in older adults. The evidence supports the feasibility and efficacy of a conservative surgical strategy, demonstrating that enterotomy alone can be a safe and effective option for high-risk patients, aligning with the principle of minimizing surgical intervention. Ultimately, this article advocates for a patient-centered approach to the management of biliary ileus, ensuring timely and appropriate interventions that reduce operative risks and enhance outcomes in vulnerable populations.

## CRediT authorship contribution statement

MEGR - Diagnosis and follow-up and Surgical approach plan.

LLMG- File tracking and documentation.

AJGL- Bibliographic review.

CVCC- Data analysis.

TSQL- Article redaction.

## Consent

Informed consent was obtained from the patient involved in this clinical case. All efforts were made to ensure confidentiality and compliance with ethical guidelines.

## Ethical approval

The present study is a presentation of a clinical case. We point out that, in our institution, it is not necessary to be submitted to or approved by an ethics committee.

## Guarantor

Quitzia Libertad Torres Salazar

## Sources of funding

This study did not receive any specific grant from funding agencies in the public, commercial, or not-for-profit sectors.

## Declaration of competing interest

The authors declare that there are no conflicts of interest regarding the publication of this article.

## References

[1] Valencia-Martínez J., Reynoso-Saldaña D., Reynoso-González R., Estrada-Hernández D., Ángeles-Santillán M., Aja-Sixto V. (2023). Gallstone ileus, a rare cause of intestinal occlusion. A case report. Cir Cir..

[2] Nik Mazian A., Ab Rahman S. (2021 Jan). Gallstone ileus. Med. J. Aust..

[3] Acevedo Forero A., Prada Rey A., Parra-Izquierdo V., Frías-Ordoñez J., Ardila-Báez M. (2024 Jan). íleo biliar como causa de obstrucción intestinal mecánica: reporte de un caso [Gallstone ileus as a cause of mechanical intestinal obstruction: a case report]. Rev. Gastroenterol. Peru.

[4] Sohrabi C, Mathew G, Maria N, Kerwan A, Franchi T, Agha R. Collaborators. The SCARE 2023 guideline: updating consensus surgical CAse REport (SCARE) guidelines. Int. J. Surg. 2023 May; 109(5): p. 1136–1140. 10.1097/JS9.0000000000000373 PMC10389401 37013953

[5] Reyes-Morales J., Hernández-García L. (2023 Jan). Gallstone ileus: a diagnostic and therapeutic challenge. Presentation of a clinical case. Rev. Med. Inst. Mex. Seguro Soc..

[6] Vera-Mansilla C., Sanchez-Gollarte A., Matias B., Mendoza-Moreno F., Díez-Alonso M., Garcia-Moreno N. (2022 Feb). Surgical treatment of gallstone ileus: less is more. Visc Med..

[7] Da Cunha T, Sharma B, Goldenberg S. Colonic Gallstone Ileus: Treatment Challenges. Cureus. 2021 Nov; 13(11): p. e19869. 10.7759/cureus.19869 PMC8709563 34963869

[8] Boudou M., Jabi R., Maamar K., Soussan H., Taibi S., Bouziane M. (2022 May). A febrile occlusion revealing a biliary ileus. Int. J. Surg. Case Rep..

[9] Helmy N., Ryska O. (2023 Jan). Gallstone ileus post-cholecystectomy: a case review. Cureus.

[10] Xue P., Zhou M., Wang Z. (2023 Jul). Duodenal gallstone ileus: a rare type of intestinal obstruction. Asian J. Surg..

